# Is mechanism of injury alone in the prehospital setting a predictor of major trauma – a review of the literature

**DOI:** 10.1186/1752-2897-1-4

**Published:** 2007-11-26

**Authors:** Malcolm J Boyle

**Affiliations:** 1 Monash University, Department of Community Emergency Health and Paramedic Practice, PO Box 527, Frankston 3199, Victoria, Australia

## Abstract

**Background:**

The literature identifying mechanism of injury came to prominence in the mid to late 1980s. The current Victorian prehospital triage guidelines do not necessarily reflect the conditions within the Victorian population as the triage guidelines are based on studies undertaken and validated in the U.S.A. The objective of this study was to identify the mechanism of injury alone literature and the predictability of the mechanism criteria.

**Methods:**

A search of the prehospital related electronic databases was undertaken utilising the Ovid and EMASE systems available through the Monash University library. The Cochrane Central Register of Controlled Trials, MEDLINE, CINAHL, and EMBASE databases were searched from their beginning until the end of June 2006. Selected non-electronic listed prehospital journals were hand searched. References from articles gathered were reviewed.

**Results:**

The electronic database search located 203 articles for review. Three additional articles were identified from the reference lists. Of these articles 17 were considered relevant. After reviewing the articles only five provided sufficient information about mechanism of injury alone and its triage capability. None of the articles identified mechanism of injury criteria as a good predictor of major trauma.

**Conclusion:**

This study identified only five articles on the predictability of the mechanism of injury criteria alone. All studies stated that the mechanism of injury criteria alone are not good predictors of major trauma or the need for trauma team activation. This study was the precursor of a Victorian prehospital study to determine the predictability of the mechanism of injury alone criteria for trauma patients in the Australian context.

## Background

The literature identifying mechanism of injury (or injury mechanism) came to prominence in the mid to late 1980s, with the mechanism of injury criteria being used as a component of triage for the trauma patient.

In the 1980s specific mechanism of injury criteria were used in conjunction with other triage scores, e.g. Injury Severity Score (ISS) and Trauma Score (TS), in assessing the severity of trauma in patients. The mechanism of injury criteria were used as an identifier of major trauma and as an indicator for transport to a trauma centre, or as a component of a trauma triage score [1-7].

In the 1990s the mechanism of injury was used as a component of a triage tool or score to determine characteristics of patients that require transport to a trauma centre [8-15]. The mechanism of injury was also used to define how the patient obtained the injury, e.g. fall > 20 feet, and not as an indication of major trauma alone. Most of these studies excluded patients over 55 years of age who had fallen and had an isolated hip fracture. A study by Esposito et al looked at mechanism of injury and also included the new criteria of "gut feeling". The analysis did demonstrate that a combination of mechanism of injury and "gut feeling" increased the prediction of major trauma patients [10]. The study by Ma et al is the only one in the 1990s that stratified each component of triage (physiological, injury pattern and mechanism of injury), again this study only used specific mechanism of injury criteria for patients transported to a trauma centre [11].

The majority of studies across both decades have treated paediatric and geriatric patients differently, some have included them both, others have not included paediatrics, and only one study has specifically investigated trauma triage in paediatrics or geriatrics [12,13].

Following the report of the Victorian Ministerial Task Force on Trauma and Emergency Services in 1999, two significant prehospital care questions remained unresolved [16]. The first, is mechanism of injury alone a useful predictor in prehospital trauma triage?, and secondly, what is the appropriate triage strategy for patients who severely deteriorate at the scene or during transport? Validation of the predictive value of the mechanistic criteria alone has been limited in Victoria and Australia whilst the international literature varies considerably.

The objective of this study was to identify the mechanism of injury alone literature and the predictability of the mechanism criteria.

## Methods

A search of the prehospital related electronic databases was undertaken utilising the Ovid and EMASE systems available through the Monash University library.

The databases searched included the Cochrane Central Register of Controlled Trials (CENTRAL), MEDLINE, CINAHL, and EMBASE from their beginning until the end of June 2006. Selected non-electronic database listed prehospital journals, where the journals were available, were hand searched.

The MeSH headings and keywords used in the searches were: ambulance, air ambulance, prehospital care, emergency medical services, emergency medical technician, out-of-hospital, out of hospital, prehospital, prehospital, paramedic, military medicine, mechanism of injury, and injury mechanism. The MeSH headings and keywords were used individually and in combination during the search process. All search results were then combined to remove duplicates and provide a list of articles for review.

The references from articles gathered were reviewed to identify additional articles not found in the electronic database and hand search.

Articles of any study type were included if they reported mechanism of injury or injury mechanism for trauma related incidents covering adult and paediatric patients in the prehospital setting. Articles were excluded if they were not written in English.

Formulas for under and over triage were derived from the article by Smith and Bartholomew [17].

## Results

The electronic database and hand search located 203 articles for review. There were three additional articles identified by reviewing the reference lists of the located articles. Of these articles 17 were considered relevant to assist in answering the research question.

After reviewing the seventeen articles considered relevant only five articles provided sufficient information about mechanism of injury alone and it's triage capability [5,9,18-20]. The remaining 12 articles contained information about mechanism of injury in the article but it was not possible to accurately separate the mechanism of injury data as it was combined with other data or the individual mechanism criteria were not defined [2,3,6,11,14,21-27].

It is difficult to compare all studies that evaluated mechanism of injury alone as the mechanism criteria varied across the studies as did the endpoints for assessing major trauma. The only consistent endpoint for assessing major trauma or severe injury was an Injury Severity Score (ISS) > 15, other endpoints varied for each study, such as hospital length of stay.

Some studies use different calculations to determine the overtriage and undertriage rates, some use specificity and sensitivity and others use positive predictive and negative predictive value. The undertriage and overtriage rates below, where the appropriate data is available, are reported using sensitivity and specificity so that comparisons can be made.

Of the five studies that separately report mechanism of injury alone not all attempted to identify major trauma as an outcome but used the criteria for trauma team activation. Likewise these studies did not report the outcome in the same manner with some of the results having to be calculated from the information contained within the article.

### Article Results

#### Long et al – 1986 [5]

The study by Long and colleagues used retrospective data from January 1983 to April 1985 in Portland, Oregon. The study involved one major hospital. The aim of the study was to evaluate the trauma score, mechanism of injury, and a combination of both scores as a triage tool to ensure that appropriate patients were transported to a trauma centre.

The study included just under 900 patients who had a trauma score determined at the incident. No specific mention was made about paediatric patients. There were 370 patients who were included in the mechanism of injury section of the study. The mechanism of injury alone criteria and results are listed in Table 1.

**Table 1:** Mechanism of Injury Only – Long et al [5]

**Criteria**	**Patients** **n**	**ISS > 16**	**Sensitivity** **%**	**Specificity** **%**
Structural intrusion into the patient's space	128	72	56	46
Motor vehicular accident with an extrication time of longer than 20 minutes	52	38	73	73
Patient ejected from the vehicle	82	53	65	65
Fall of 15 ft (4.6 m) or more	37	22	59	59
Other loss of life in the same vehicle compartment	25	21	84	84
Child less than 12 years of age struck by car	18	7	39	39
Pedestrian struck by vehicle and thrown	28	18	64	64

**Figure 1:**
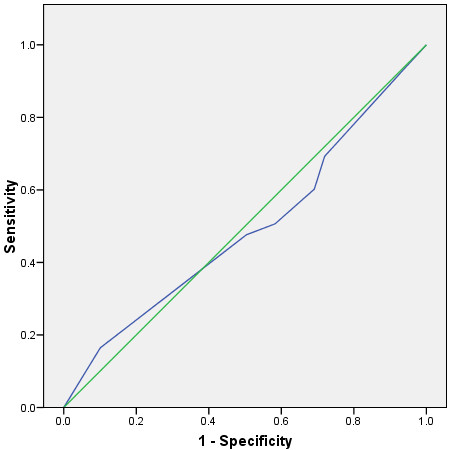
ROC Curve for Long et al Mechanism of Injury and Major Trauma.

As the results demonstrate the overtriage rate varies between 16% and 61%, with the undertriage rate between 16% and 61%. The area under the ROC Curve was 0.49 (95% CI 0.43 to 0.55, p = 0.8), see Figure 1. Long et al found, like other authors on the topic, when combining the mechanism of injury criteria with the trauma score it decreased the overtriage and undertriage of patients.

#### Knudson et al – 1988 [18]

The study by Knudson and colleagues looked at 500 consecutive patients presenting to a trauma centre in Santa Clara Country in California during 1984. The aim of the study was to determine if prehospital triage criteria was able to identify trauma patients with serious injuries that presented to the trauma centre.

There was no comment about the inclusion or exclusion of paediatric patients. Sensitivity and specificity analysis of the data was undertaken so that comparisons could be made between the triage criteria and their ability to predict patients with serious injuries. Even though Knudson et al did not report the undertriage and overtriage results they have been calculated. The mechanism of injury alone criteria and results are listed in Table 2.

**Table 2:** Mechanism of Injury Only – Knudson et al [18]

**Criteria**	**Patients** **n**	**Sensitivity** **%**	**Specificity** **%**
Fall > 16 feet (4.9 m)	33	4	96
Motorcycle accident > 20 mph (32 km/h)	39	9	94
Automobile versus pedestrian accident > 5 mph (8 km/h)	78	16	81
Rollover accident	27	5	94
Motor vehicle accident > 40 mph (64 km/h)	126	24	72
Bicycle accident	12	0	97
Death of Passenger and Prolonged Extrication	41	NS	NS

NS: Not Specified in the article.

Knudson et al found that mechanism of injury criteria alone were not good predictors of serious injury, but when the mechanism of injury criteria were combined with the Trauma Score [28] and CRAMS [29] score the predictability improved to an acceptable level. As can be seen in table 2, the best predictor of the mechanism criteria was motor vehicle accident > 40 mph (64 km/h) at 24%.

There is insufficient data in the article to calculate the sensitivity and specificity for the Death of Passenger and Prolonged Extrication criteria. The undertriage rates in this study ranged from 76% to 96%, with the overtriage rates ranging from 4% to 28%. The author stated in the conclusion that they were willing to accept an overtriage rate of 60% in order to achieve an undertriage rate less than 10%.

#### Simon et al – 1994 [19]

The study by Simon and colleagues looked at twelve months of retrospective data for patients who presented to a trauma centre between July 1992 and July 1993. The aim of the study was to assess the criteria for trauma team activation.

Simon et al retrospectively categorised the patients into two groups, those with minor injuries and those with potentially severe injury. Paediatric patients, aged less than 16 years, were excluded from the review.

The study group produced a Vehicular Trauma Checklist to assist in categorising the patients on arrival at the emergency department to minor injury or potentially severe injury. The study found 347 patients with mechanism of injury alone, the mechanism criteria and results are listed in Table 3.

**Table 3:** Mechanism of Injury Only – Simon et al [19]

**Criteria**	**Patients** **n**	**Severe** **Injury**	**Sensitivity** **%**	**Specificity** **%**
Rollover	59	15	25	59
Head-on > 30 mph (48 km/h)	130	50	38	61
Ejected	11	5	45	62
Intrusion	130	53	41	63
Prolonged extrication	17	11	65	63

Severe Injury is any one of the following criteria: hypotension; urgent interventions in the trauma room (airway control, chest tube, transfusion, diagnostic peritoneal lavage, central venous catheter or venous cutdown); transfer to ICU or OR; and an ISS > 5.

**Figure 2:**
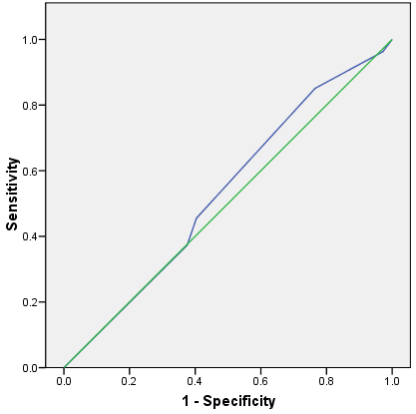
ROC Curve for Simon et al Mechanism of Injury and Trauma Team Activation.

Simon et al reported that 38% of the trauma patients triaged to the trauma team actually had serious injury. Using the Vehicular Trauma Checklist tool they created it increased to 61% of the patients. The area under the ROC Curve was 0.53 (95% CI 0.47 to 0.59, p = 0.3), see Figure 2. The overtriage rates in this study for mechanism of injury alone ranged from 37% to 41%, with the undertriage rates ranging from 35% to 75%.

#### Bond et al – 1997 [9]

The study by Bond and colleagues looked at a six month period from May to October 1995 in Calgary, Canada. Bond et al looked at trauma triage scoring to determine which patients required transport to a designated trauma centre for their on going management. The criteria for major trauma was an ISS score of > 15. This study did not included children less than 15 years of age. The mechanism of injury alone criteria and results are listed in Table 4.

**Table 4:** Mechanism of Injury Only – Bond et al [9]

**Criteria**	**PPV – %**
Extrication time > 20 minutes	40.0
motorcycle crash victims ejected at greater than 30 km/h	22.4
Motor vehicle crash: death or serious injury to other occupant in the same car	21.4
Motor vehicle crash: a steering wheel deformity or structural intrusion into the passenger compartment of greater than 20 inches (508 mm)	17.9
Fall > 15 ft (4.6 m), with head involvement, or falls that occur on staircases	14.3

The study reported an undertriage rate of 27% for all mechanisms of injury and an overtriage rate of 9%. The study did not report individual mechanism criteria results but an overall mechanism result. There were no patient numbers listed in the article for each mechanism of injury criteria. There was no predictive value reported for a pedestrian struck at a velocity of greater than 15 km/h.

The mechanism of injury alone results were:

• sensitivity – 73%

• specificity – 91%

• positive predictive value – 18%

• negative predictive value – 99%

This study did not report results for the mechanisms of injury criteria defined in the results section of the article but reported on another set of mechanism criteria. It is therefore difficult to know whether the reported mechanisms produced a different set of results compared to the mechanisms defined at the outset of the project as there were no results reported for these criteria.

#### Qazi et al – 1998 [20]

The study by Qazi and colleagues look specifically at a paediatric population between July 1993 and July 1994 at a paediatric trauma centre in Akron, Ohio, U.S.A. The aim of the study was to determine the ability of mechanism of injury to identify major trauma in stable paediatric patients, trauma team activation was also based on this criteria.

The mechanisms of injury used in the study were:

• motor vehicle accident

• passenger in a motor vehicle accident

• ejected from vehicle

• rollover motor vehicle accident

• pedestrian struck by vehicle travelling > 10 mph (16 km/h)

• motor vehicle accident with death of another occupant

• bike accidents

• falls > 10 feet (3 m)

The study found 194 patients that met their criteria with 143 patients presenting with mechanism of injury criteria only. The patient numbers for each mechanism criteria was not listed in the article. There was insufficient information to calculate the sensitivity and specificity for each mechanism criteria. The authors reported a sensitivity of 44.4% and a specificity of 24.9%, a positive predictive value of 2.8% and a negative predictive value of 90.2%. These results demonstrate an overtriage rate of 75% and an undertriage rate of 56%, the authors state that mechanism of injury alone criteria are not useful predictors of major trauma and are not useful, by themselves, for trauma team activation.

## Discussion

This is the first paper to specifically identify studies of trauma patients, of all ages, with a mechanism of injury alone, and the predictability of major trauma for these mechanisms. The literature search and review located only five relevant studies that investigated the mechanism of injury alone criteria and their ability to predict major trauma or the need for trauma team activation.

The literature identifying mechanism of injury (or injury mechanism) came to prominence in the mid to late 1980s, with the mechanism of injury being used as a component of triage for the trauma patient. The current Victorian prehospital triage guidelines have been in place since mid the 1980's and have undergone little, if any, significant change over that time. These triage guidelines do not necessarily reflect the conditions within the Victorian setting as the triage guidelines are based on studies undertaken in the U.S.A. One should therefore take into account the differences between the respective health care systems. The current prehospital triage criteria, as a result of the Review of Trauma and Emergency Services in Victoria (ROTESV) [16], mirror closely the American College of Surgeons (ACS) triage criteria [30].

Until recent times under triage has not been a significant problem, however, with hospital emergency departments suffering from overcrowding, and in some circumstance being on ambulance bypass, there is a need for more accurate triage criteria so that trauma patients with the greatest need are delivered to the most appropriate trauma service to best manage their condition. In some cases this may not be a Major Trauma Service or Regional Trauma Service hospital but one of the other hospitals within the trauma system who can manage patients with mechanism of injury alone trauma.

Studies investigating paediatric trauma triage noted that using adult based triage criteria results in higher under triage rates in the paediatric group [22,31]. Similarly, studies investigating trauma triage in elderly patients noted that elderly patients were more likely to be significantly under triaged compared to other adults [11,15,32].

Most of the studies located looked at either motor vehicle accidents (MVA) and blunt trauma only or a small subset of trauma causes, caused by blunt trauma, for their data comparisons. Very few studies looked at all forms of trauma, as identified in the ROTESV report [16].

It is difficult to accurately compare the outcomes from all the studies that evaluated mechanism of injury alone as the mechanism criteria varied across the studies as did the endpoints for assessing major trauma. The only consistent endpoint for assessing major trauma or severe injury was an ISS score >15, other endpoints varied for each study, such as hospital length of stay. The studies predominately used data from a limited source, e.g. one trauma centre or hospital, therefore limiting the external validity of the study.

Of the five studies that separately reported mechanism of injury alone, not all these studies attempted to identify major trauma as such but used the criteria for trauma team activation. Likewise these studies did not report the outcome in the same manner with some of the results having to be calculated from the information contained within the article. In an attempt to enable a meaningful comparison of these studies, an attempt has been made to use one set of criteria, namely sensitivity and specificity. Recalculation, using data from the original articles has been possible, and as far as is possible, enables these studies to be compared.

The five studies collectively suggest that the mechanism of injury criteria alone are not useful predictors of major trauma. However, all the results relate to small study numbers, especially for each mechanism criteria, therefore the results should be interpreted with caution. Shatney et al found that using mechanism of injury alone as a criterion for trauma team activation was a waste of resources, 75% of these patients were discharged home from the emergency department, 26% were admitted to hospital, and only 0.17% required early surgery (less than 12 hours) [24].

The study by Palanca was the only Victorian study identified that looked at mechanism of injury, however, this was in the review of mechanism criteria that lead to major trauma. There was no mechanism of injury alone data that could be extracted from the study data [25].

The evidence presented suggests that using the same mechanism of injury in Victoria that were identified in the located studies would more than likely produce similar results of under/overtriage. Several of the studies did demonstrate that when the mechanism of injury criteria is combined with a prehospital or similar trauma triage tool that the accuracy of the tool increased. However, there are still a large number of patients that are overtriaged and undertriaged. The elderly (those greater than 55 years of age), and some paediatric patients are more likely to be undertriaged than the general trauma population. Criteria using physiological, injury components, and mechanism of injury are more likely to better identify potential major trauma patients but also have a high over triage rate.

This study was the precursor of a Victorian based prehospital trauma study to establish the predictability of the mechanism of injury alone criteria in the Australian healthcare context. The results from U.S.A. based studies, in some cases, are old, and were undertaken in a vastly different healthcare system.

This study is potentially limited in that it did not include non English studies in the search criteria and subsequent review.

## Conclusion

This study demonstrates that there have been few studies into mechanism of injury criteria alone and their predictability of major trauma. The studies all stated, and the evidence supports, that the mechanism of injury criteria alone are not good predictors of major trauma or the need for trauma team activation. There is a need for an Australia based trauma study investigating the mechanism of injury alone criteria and their ability to predict major trauma.

## Competing interests

The author(s) declare that they have no competing interests.
